# The use of yoga and mindfulness within an eating disorders population: Results of a scoping review

**DOI:** 10.1192/j.eurpsy.2021.957

**Published:** 2021-08-13

**Authors:** F. Dontigny, C. Debreucker, E. Therrien, J. Monthuy-Blanc

**Affiliations:** 1 Biomedical Sciences, Université du Québec à Trois-Rivières, Trois-Rivières, Canada; 2 Programme Loricorps, Université du Québec à Trois-Rivières, Trois-Rivières, Canada; 3 Education, Université du Québec à Trois-Rivières, Trois-Rivières, Canada

**Keywords:** eating disorders, yoga, mindfulness, integrative health

## Abstract

**Introduction:**

Eating disorders (ED) are characterized by perturbed eating habits or behaviors (APA, 2013). Even if treatments are available, they need to be more adapted to ED (Monthuy-Blanc, 2018). A complementary approach as yoga or mindfulness demonstrated positive effects with ED, such as an augmentation of mindfulness while eating (Rachel, Ivanka, Amanda, & Carlene, 2013), a better body satisfaction (Beccia, Dunlap, Hanes, Courneene, & Zwickey, 2018; Neumark-Sztainer, MacLehose, Watts, Pacanowski, & Eisenberg, 2018) and less preoccupation with food (Carei, Fyfe-Johnson, Breuner, & Brown, 2010). As the effects of yoga and mindfulness vary between the different ED and different uses, it is difficult to generalize the results obtained about the efficacy of yoga or mindfulness with ED.

**Objectives:**

A scoping review is actually done to map the evidence about the use (length, intensity, frequency) of yoga and mindfulness among ED and their effects.

**Methods:**

The realization of the scoping review is based on the Joanna Briggs Institute Methodological Framework(Peters, Godfrey, McInerney, Baldini Soares, Khalil, & Parker, 2017). Research will be done in the following databases: CINAHL, PsycInfo, PubMed/MEDLINE, Web of Science, EBM Reviews/Cochrane. Different types of papers are going to be included and a content analysis is going to be done among the extracted data.

**Results:**

Preliminary results of the scoping review are going to be presented.

**Conclusions:**

Among the different treatments used with ED, yoga and mindfulness have demonstrated positive effects. These approaches as part of integrative health are helpful to improve physical and mental health of individuals suffering from ED.

